# Skin microdialysis: methods, applications and future opportunities—an EAACI position paper

**DOI:** 10.1186/s13601-019-0262-y

**Published:** 2019-04-10

**Authors:** Katrine Y. Baumann, Martin K. Church, Geraldine F. Clough, Sven Roy Quist, Martin Schmelz, Per Stahl Skov, Chris D. Anderson, Line Kring Tannert, Ana Maria Giménez-Arnau, Stefan Frischbutter, Jörg Scheffel, Marcus Maurer

**Affiliations:** 1 RefLab ApS, Copenhagen, Denmark; 2 Department of Immunology and Microbiology, University of Copenhagen, Copenhagen, Denmark; 3 Department of Dermatology and Allergy, Charité – Universitätsmedizin Berlin, Charitéplatz 1, 10117 Berlin, Germany; 4 Faculty of Medicine, University of Southampton, Southampton, UK; 5 Clinic of Dermatology, Otto-von-Guericke University, Magdeburg, Germany; 6 Skin Center MDZ, Mainz, Germany; 7 Department of Experimental Pain Research, CBTM, Medical Faculty Mannheim, Heidelberg University, Heidelberg, Germany; 8 Odense Research Center for Anaphylaxis (ORCA), Department of Dermatology and Allergy Center, Odense University Hospital, Odense, Denmark; 9 Department of Clinical and Experimental Medicine, Linköping University, Linköping, Sweden; 10 Department of Dermatology, Hospital del Mar, Institut Mar d’Investigacions Mèdiques, Universitat Autònoma, Barcelona, Spain

**Keywords:** Microdialysis, Cutaneous, Inflammation, Interstitial, Dermal

## Abstract

Skin microdialysis (SMD) is a versatile sampling technique that can be used to recover soluble endogenous and exogenous molecules from the extracellular compartment of human skin. Due to its minimally invasive character, SMD can be applied in both clinical and preclinical settings. Despite being available since the 1990s, the technique has still not reached its full potential use as a tool to explore pathophysiological mechanisms of allergic and inflammatory reactions in the skin. Therefore, an EAACI Task Force on SMD was formed to disseminate knowledge about the technique and its many applications. This position paper from the task force provides an overview of the current use of SMD in the investigation of the pathogenesis of chronic inflammatory skin diseases, such as atopic dermatitis, chronic urticaria, psoriasis, and in studies of cutaneous events during type 1 hypersensitivity reactions. Furthermore, this paper covers drug hypersensitivity, UVB-induced- and neurogenic inflammation, and drug penetration investigated by SMD. The aim of this paper is to encourage the use of SMD and to make the technique easily accessible by providing an overview of methodology and applications, supported by standardized operating procedures for SMD in vivo and ex vivo.

**Electronic supplementary material:**

The online version of this article (10.1186/s13601-019-0262-y) contains supplementary material, which is available to authorized users.

## What is skin microdialysis?

**Fig. 1:**
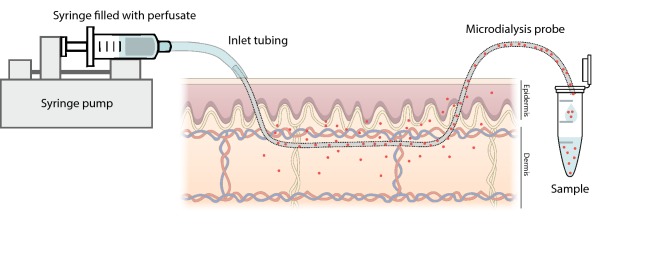
Schematic representation of SMD (here with a linear microdialysis probe). The membrane is inserted into the tissue from which it allows recovery of soluble molecules (in red) when perfused using a microperfusion pump. © Niels Peter Hell Knudsen

To perform skin microdialysis (SMD) thin tubular dialysis membranes are inserted into the dermis or the subcutis and perfused at a low speed with a physiological solution (the perfusate) (Fig. 1). Endogenous or exogenous molecules soluble in the extracellular fluid diffuse into the tubular microdialysis membrane and are collected in small vials for analysis. The duration and timing of the collected dialysate samples allows kinetic evaluation of the events occurring in the tissue.

In broad terms, microdialysis has been applied in two scenarios. The first and in fact the original use of the technique aimed to gain continual, real-time data reflecting target tissue status as an alternative to repeated blood sampling. This monitoring situation usually, because of the insertion technique used, involved placement of probes in the subcutaneous layer of the skin. It allowed early detection of a metabolic deterioration in, for instance, an intensive care patient with sepsis. With time, the technique began to be used for specific studies elucidating the role of the actual subcutaneous tissue [1]. Specific placement of probes into the dermal layer opened the way for studies of inflammatory events most prominently driven by that part of the skin. SMD has also been applied in drug discovery and pharmacokinetic/pharmacodynamic (PK/PD) studies (for reviews see [2–4]) and in the study of percutaneous penetration of potentially harmful exogenous agents from the environment [5]. SMD has the advantage over other tissue sampling techniques of being minimally invasive, and it is well tolerated by human volunteers. As a consequence it has been widely used to study normal, diseased and experimentally perturbed skin function [6–8]. In the 25 years since the introduction of the technique, over 800 papers have been published on SMD.

The purpose of this paper is to review how the use of SMD has helped to improve our understanding of chronic inflammatory skin conditions and skin inflammation in general. We also hope to encourage the use of SMD for investigating the many remaining unanswered questions on the mechanisms of cutaneous inflammation especially in the field of skin allergy and skin hypersensitivity reactions.

## How SMD has helped us to understand skin inflammation and skin inflammatory disorders

### What SMD taught us about cutaneous type 1 hypersensitivity reactions

The wheal and flare response to dermal provocation with allergen is a well-established model of type 1 hypersensitivity. SMD is an ideal technique with which to investigate the mechanisms of this response by identification of the inflammatory mediators generated in vivo in real-time [9–11].

The mechanism of the early phase response has been investigated by insertion of microdialysis probes into different areas of the wheal and flare [12]. The results showed that histamine was released in the wheal but not the flare. Further studies [13] showed that the neurogenic flare was mediated primarily by calcitonin gene related peptide (CGRP).

The use of scanning laser Doppler imaging in combination with SMD has allowed the investigation of quantitative real-time temporal and spatial changes in blood flow in response to other potential inflammatory mediators in the skin. For example, the H_1_-antihistamines cetirizine and loratadine were shown to inhibit wheal and flare responses to bradykinin as well as histamine [14]. The obvious conclusion from this study was that bradykinin induces histamine release, particularly as cetirizine was shown not to interact with either the B_1_ or B_2_ bradykinin receptors [15]. However, microdialysis showed that this was not the case in most individuals [14]. Instead, the results of the use of SMD suggest that there is co-operativity between bradykinin and histamine H_1_-receptors in the dermis. A similar scenario has been found with platelet activating factor [16].

In a further study [7], the cytokine response to dermal allergen provocation was studied in 11 allergic individuals over a period of 6 h using two linear SMD probes inserted 1 cm apart in the volar skin of the forearm. Allergen injection caused a significant rise in interleukin (IL)-6 within 30 min. However, increased tumor necrosis factor (TNF)-α levels were found in only 3 individuals at this time. At both 3 and 6 h, significantly elevated levels of IL-1α, IL-1β, IL-6 and IL-8 were found. Interestingly, IL-6 and IL-8 were also raised at the site 1 cm from the allergen injection. In contrast, adhesion molecule expression and leukocyte infiltration were elevated only at the allergen injection site, suggesting a complex relationship between cytokine generation and cellular events in allergic inflammation. A further fascinating outcome of this study was that, when looked at individually, the cytokine profile of every person was different illustrating the need for further human SMD studies to unravel the complexities of immunological skin responses.

### How SMD has helped our understanding of atopic dermatitis

The particular strength of SMD in studies of atopic dermatitis (AD) is combining analysis of local mediator concentrations with the assessment of sensory perception. Intra-probe delivery of mast cell-degranulating codeine provokes local wheal and C-fiber activation resulting in an axon reflex erythema and histamine-independent itch in patients with AD [17]. This response is mediated probably via increased mast cell tryptase activating nociceptors via proteinase-activated receptors [17, 18]. Higher iron and ascorbic acid as wells as prostanoid levels were found in the skin of AD patients [19, 20] whereas no significant increase in nerve growth factor was detected [21]. Intra-probe delivery of prostaglandin (PG)E2 [22] and low pH perfusate [23] were successfully used to show the sensitized neuronal itch response to normally painful stimuli in patients with AD.

In pain research, SMD has been used to assess the link between local mediator release and neuronal sensitization in more detail [24, 25]. Thus, using improved analytical methods, SMD will successfully identify clinically relevant local mediator concentrations in AD such as large signaling peptides, local hormones and lipids.

### Insights from SMD studies on psoriasis

Cytokine profiles of SMD-derived samples analyzed by bead-based multiplex immunoassays have been used to monitor changes in the micromilieu of the skin of patients with psoriasis for up to 24 h. Post-equilibration levels at 17–24 h showed that granulocyte-macrophage colony-stimulating factor (GM-CSF) and TNF-α levels were elevated in psoriasis compared with healthy subjects [26]. In another study, levels for IL-2, IL-6, IL-18 and IL-23 were elevated in dialysates of lesional versus non-lesional skin prior to therapy. Clinical improvement under 12 weeks of continuous oral therapy with fumaric acid esters paralleled the reduced concentrations of these cytokines in dialysates [27]. The same group reported that IL-1β was elevated in dialysates from psoriasis plaques compared with non-lesional skin, and levels were reduced under successful anti-psoriatic fumaric acid esters therapy [28]. A pharmacokinetic profile was elaborated in patients with psoriasis using SMD comparing lesional and non-lesional skin with intravenous microdialysis after administration of oral or subcutaneous methotrexate. Methotrexate bioavailability was higher in psoriasis plaques than in non-lesional skin but highly individual [29]. Several SMD studies analyzed histamine release examined by high-performance liquid chromatography (HPLC) in psoriatic skin and showed a tenfold increase in lesional compared to non-lesional skin [30]. Ranitidine was able to reduce histamine release in lesional skin [31].

### Chronic urticaria: what did we learn from SMD studies?

SMD is ideally suited for the investigation of inducible urticaria, because its signs and symptoms (itchy wheals and angioedema) can be induced by skin provocation with relevant triggers. Most SMD studies have investigated cold urticaria, first in 1995 when histamine release was demonstrated in wheals elicited by an ice cube test in cold urticaria patients [32]. Nuutinen et al. reported similar results [33] but failed to demonstrate leukotriene C_4_ (LTC_4_) release. They concluded that the absence of LTC_4_ could be due to the activation of skin mast cells by an IgE-independent mechanism. Taskila et al. also failed to detect LTC_4_ by SMD in volunteers challenged with stinging nettles [34]. In contrast, Horsmanheimo et al., also using SMD, measured increase of LTC_4_ in volunteers after controlled challenge with mosquito bites [35].

SMD has also been used to monitor the therapeutic effect of desensitization or antihistamines in cold urticaria patients by measuring histamine or cytokine release in response to cold provocation. For example, Tannert et al. investigated cold desensitization in cold urticaria patients [36] and found, before desensitization, histamine release by SMD in wheals elicited by cold challenge but no histamine release upon a subsequent codeine skin test. After successful desensitization, no histamine was released at cold-exposed skin sites while codeine challenge resulted in histamine release indicating that the mechanism of cold desensitization is unlikely to be due to depletion of histamine in skin mast cells. In a study by Krause et al., the beneficial effect of using increased doses of the non-sedating antihistamine bilastine in patients with cold urticaria was shown [37]. SMD in cold challenged patients with cold urticaria treated with increased doses of bilastine showed significantly reduced late phase histamine and proinflammatory cytokine (IL-6 and IL-8) release.

### The use of SMD in studies of drug hypersensitivity and ultraviolet B (UVB)-induced skin responses

Skin provocation testing with drugs or UVB radiation allows for assessing skin responses by SMD, for example to sample the real-time release of biomarkers. For drug hypersensitivity studies, the skin can be challenged directly by performing skin tests with the drug close to the probe to elicit a wheal that develops across the probe. Experimentally, the impact of treatment on mediator release can be studied and compared to placebo treatment. While SMD is well suited for assessing drugs that elicit immediate reactions in the skin, delayed drug reactions mediated by T cell activation are more challenging to study by SMD. Nevertheless, SMD is promising, because mediators of different sizes can be sampled by use of probes with varying molecular weight cut-off (MWCO). So far, SMD has been used to a limited extent in the investigation of drug hypersensitivity. In one study, SMD in penicillin allergic patients demonstrated that histamine was only released in the minority of positive intracutaneous tests with penicillin [38].

SMD has been used in several studies of the release of prostanoids and cytokines following UVB radiation [24, 39–42]. In one study, SMD was performed 24 h before and 24 h after UVB challenge, and dialysates were sampled at 8-h intervals [39]. Probe placement 24 h prior to radiation induced an unspecific proinflammatory, traumatic response driven by IL-6, IL-8, TNF-α and IL-1β, whereas UVB radiation showed a mixed T_H_1/T_H_2-related cytokine profile, with a late IL-4 and IL-10 dominant T_H_2-driven shift. A more recent SMD study elaborated a kinetic profile for inflammation markers 16 h prior and 48 h post radiation [41]. Dialysates were collected at 4-h intervals and analyzed for 5- and 8-iso-PGF_2α_, 9α,11α-PGF_2α_ and PGE_2_ using gas-chromatography/mass-spectrometry and for IL-1β, IL-2, IL-3, IL-4, IL-5, IL-6, IL-8, IL-10, TNF-α, Fas ligand (FasL), interferon-γ-induced protein 10 (IP-10), monocyte chemoattractant protein 1 (MCP-1), regulated on activation normal T cell expressed and secreted (RANTES), eotaxin, and GM-CSF using a multiplex cytometric-bead-array. As a result, 3 peaks with synchronic release of T_H_1-directed inflammatory cytokines and prostanoids could be detected post-UVB radiation: an early phase (4–12 h), an intermediate phase (16–24 h) and a late phase (32–40 h). A T_H_2-directed cytokine response was detectable during intermediate and late phases. This study indicated that the release of cytokines and prostanoids is synchronized and that a slow T_H_1-to-T_H_2 shift occurs up to 40 h after UVB radiation.

### The use of SMD to study neurogenic inflammation

The activation of peptidergic nociceptors in the skin causes the release of neuropeptides that dilate precapillary arterioles (calcitonin-gene related peptide, CGRP) and increase leakiness of post-capillary venules (substance P, SP), i.e. neurogenic inflammation. SMD has been used to apply neuropeptides and assess dose–response functions for neurogenic inflammation and itch including studies that suggest a role of nitric oxide in neurogenic vasodilation in human skin [43]. While histamine release following nociceptor activation has been shown in rodent skin [44], this is not the case in humans within the axon reflex flare area [45]. SP-release in humans is less pronounced as compared with rodents and there is no neurogenic protein extravasation in healthy volunteers [46, 47]. However, there are chronic pain conditions in which SP-upregulation might enable neurogenic protein extravasation also in humans [48] even in the non-affected limb [49, 50]. More tardy neuropeptide degradation increases neurogenic vasodilation [51] and might be of clinical importance in chronic pain conditions.

## How SMD is used to study drug penetration and distribution

Following on the use of SMD to investigate metabolic events in the human body, the study of percutaneous penetration of exogenous substances has been arguably the first dermatological area studied by microdialysis [52]. Several ways of delivery to the skin of drugs and other agents of interest have been studied [5, 53, 54], and SMD has also been used in animal models and ex vivo models. Topics for discussion and development have clustered around membrane permeability and the “stickiness” of molecules, the analytical sensitivity required and issues of lipophilicity and tissue binding of individual target molecules. Several useful reviews are available illustrating important, generic methodological issues (e.g. [2, 55–59]). Attempts to fulfill the developmental and regulatory needs concerning bioavailability and bioequivalence of topical pharmaceuticals have, over the last two decades, involved the use of either tape stripping (so called DPK—dermato-pharmaco-kinetic modelling) or SMD, with the current emphasis on the latter. In vivo protocols involving SMD have been suggested with numbers of subjects (and thus costs) that are far lower than the traditional clinical trial methodology, which has previously been necessary to demonstrate bioequivalence of a new topical pharmaceutical product. More recently, the open flow variant of SMD involving outer membranes that are fenestrated rather than being reliant on pores, has been the subject of intensive development of technique, application and data interpretation [60, 61]. The developments have been necessary in order to standardize potential sources of variability in data such as blood flow and other interindividual variability. Since both standard SMD and open flow microperfusion have been used to demonstrate the chronology of expression of inflammatory and other tissue indicators, the integration of pharmacokinetic and pharmacological data seems entirely possible and logical.

The study of penetration of harmful agents into the skin is also possible. The microdosing nature of microdialysis (low concentrations and small areas of skin for provocation rather than larger areas of skin or systemic administration) is an important ethical advantage for studies on e.g. percutaneous penetration of pesticides or similar potentially toxic agents. In extension, SMD may have uses in studies of dose (dermal delivery) of allergens and even of their fate (by metabolism) in living skin.

## SMD techniques and methodology

### In vivo SMD

There is an extensive and wide ranging technical literature on SMD that focusses on key methodological considerations. The most important of these relate to choice of skin site, probe selection and insertion, and to probe perfusate and perfusion rate [62].

**Fig. 2:**
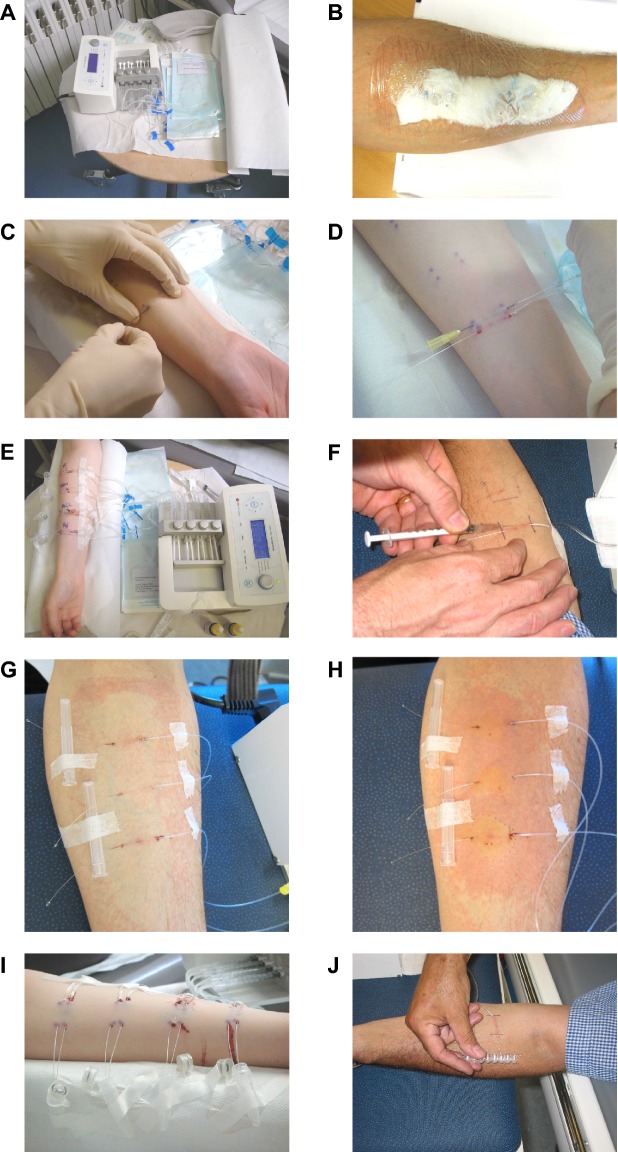
An example of the SMD procedure. **a** Priming of microdialysis probes prior to insertion, **b** topical application of local anesthesia, **c** intradermal insertion of guide cannulas, **d** introduction of probes through the guide cannulas, **e** SMD setting and basal sampling, **f** skin challenge, **g** skin site before challenge, **h** wheal and flare reaction in response to intradermal challenge, **i** collection of dialysates—here in microtubes, **j** alternative collection of dialysates—here directly into sampling wells. Please refer to the SOPs (in vivo SMD SOP and ex vivo SMD SOP, provided as Additional files 1 and 2, respectively) for a detailed description of the SMD procedure. Pictures are reproduced with kind permission from Line Kring Tannert and Marcus Maurer

The most frequently used site for in vivo SMD is the volar forearm [5, 16, 63–65] (Fig. 2), although other sites have been used to study regional variations in skin responses [66], pruritic responses (e.g. in the scalp [67]), and in the assessment of skin graft and flap viability [68]. When using the forearm, usually only one arm is used at a time to allow the participant some freedom of movement.

Probe selection is driven by the physicochemical properties of the analyte recovered; its size, charge and hydrophilicity determining the MWCO of the dialysis membrane as well as the construction of the probe itself. Linear probe membranes have a smaller diameter than the larger concentric probes, which are typically used for systemic drug delivery studies. As a result, narrow insertion needles are used for linear probes, which cause less insertion trauma. Insertion of concentric probes, on the other hand, requires only one penetration of the skin.

It is important to acknowledge that most, but not all, in vivo human SMD studies use local anesthetic. This has the advantage of reducing the pain of probe insertion (and encouraging study participation) and limiting the wounding response. However, its long action may confound studies in which changes in local blood flow and/or neurogenic responses are of interest or where they may influence the interpretation of pharmacological studies.

There are very few reports about the time necessary for recovery from trauma associated with probe insertion or about the specific endogenous compounds generated as a result of this trauma. A 2 h recovery period is usually adopted to allow local blood flow to return to normal (indicating the resolution of the immediate erythematous response to trauma) [69, 70] or a normal flare response to histamine to be re-established (indicating the recovery from the local anesthetic) [71].

Selection of perfusion medium (usually isotonic saline without or with additives to aid analyte recovery) and rate of perfusion are driven by the nature of the solute to be recovered and by the study aims (see Table 1). Volume requirements of the assay platform are also highly influential in determining probe perfusion rates and dialysate collection protocols. The recent development of microfluidic platforms for the continuous on-line sampling of dialysate may go some way towards addressing this in future [72, 73].

**Table 1:** Factors affecting recovery of analytes from the skin by microdialysis

Factors influencing analyte recovery	Effect	Recommendations/considerations	References
Analyte-related			
Molecular weight, shape and solubility	The size and water solubility of the analyte affect its diffusion through the microdialysis membrane as well as analyte diffusion in the tissue environment. Small molecules are easily recovered whereas high molecular weight molecules are more difficult to sample	In order to recover large molecules high MWCO probes must be used and the microdialysis setup should be carefully optimized (refer to the parameters listed in this table) to obtain the highest relative recovery possible	[86–88]
Molecular stability	Analyte stability in dialysates is important to consider for optimal storage and subsequent analyte detection in the samples	A refrigerated fraction collector can be used during microdialysis sampling. Dialysates containing labile analytes should be stored accordingly (e.g. at − 80 °C)	[89]
Other physicochemical properties (e.g. lipophilicity)	The physicochemical properties of an analyte affect its adherence to the tissue environment (e.g. the extracellular matrix) and the probe components. Such adherence will diminish the fraction of soluble analyte and thus analyte recovery	To improve recovery of highly lipophilic analytes a lipid emulsion can be used for perfusion. Non-specific adsorption can be decreased by adding a blocking-protein such as albumin to the perfusate	[86, 88, 90–93]

Technical			
Perfusion flow rate	The in vitro recovery is inversely dependent on the flow rate as this affects the extent of equilibrium established across the semi-permeable membrane	The flow rate should be chosen based on the target analyte(s) and the volume requirement of subsequent analyses For small molecules: 1–5 µl/min For macromolecules: < 1.0 µl/min	[82, 86, 87, 89, 94]
Sampling intervals	The length and number of sampling intervals affect the temporal resolution and may also affect the molecular stability of the analyte depending on the collection procedure	The sampling interval should be set based on a combination of the volume requirements of subsequent analyses, the temporal resolution required and the analyte stability	[88, 89]
Membrane molecular weight cut-off	The membrane cut-off is defined as the molecular weight at which 80% of the molecules are unable to pass the membrane, therefore it is not an absolute measure. It relates to the membrane pore size and thus has a great impact on analyte recovery, which is (in part) correlated to its size and shape	The optimal molecular cut-off the microdialysis membrane is partly determined by the molecular weight of the analyte but also the requirement for sample purity. Since differences in membrane material will affect the recovery, more probe types should be tested	[94–96]
Probe type	Microdialysis probes used in the skin are usually of the linear or the concentric type. The probe construction determines the maximal membrane area available for diffusion. Furthermore, the design affects the outer probe diameter and the number of penetration sites required for insertion, thus the degree of tissue trauma induced by probe insertion. Linear probes penetrate the skin twice and have a smaller outer diameter in contrast to concentric probes, which penetrates the skin once, as the inlet and outlet are placed in parallel, but at the cost of a larger outer diameter	The choice of probe type relates to commercial availability and to the anatomical site to be sampled. The degree of insertion trauma induced must be considered and so must the potential discomfort for human subjects participating in in vivo studies Linear probes can either be purchased or self-made in the lab. Self-fabrication of probes allows for customization of the membrane length and material	[58, 94, 97]
Probe/membrane material	The probe materials (including the membrane composition) affect potential non-specific adsorption of molecules to probe components as well as analyte interaction with the membrane. This is often an issue for lipophilic molecules	Inert probe materials should preferably be used. Different membrane- and tubing material can be tested with respect to diffusion of molecules across the membrane and the degree of non-specific adsorption	[89, 98, 99]
Membrane length/surface area	The analyte recovery increases with increasing membrane surface area available for diffusion	In general, the membrane length should be maximized (e.g. spanning 2 cm intradermally). However, it must be adjusted to the tissue in which the sampling is carried out	[89, 94, 98]
Perfusate composition	The composition of the perfusion medium affects recovery of molecules and water movement across the probe	A physiological solution is generally used. Additives such as albumin or dextran might improve analyte recovery and stability, while preventing fluid leakage from the probe (a frequent issue for probes with a high molecular weight cut-off) and decrease non-specific adsorption to probe components	[89, 91, 93, 94, 98, 100–102]
Temperature	In theory, diffusion increases with temperature, which can lead to a higher recovery. However, the physicochemical properties of the analyte (especially for proteins) might influence the temperature dependency	The temperature in vivo is determined by the target tissue but can be manipulated in ex vivo and in vitro experiments. In vitro validation studies should reflect the temperature of the end setup (e.g. be adjusted to body temperature)	[89, 98, 103]

Biological			
Tissue characteristics	The tortuosity of the tissue fluid space will affect analyte diffusion and therefore the recovery. Furthermore, the tissue metabolism, degree of vascularization as well as cell internalization of the analyte will affect its recovery	To obtain valid results from probe calibration studies these should be carried out in a matrix representing the tissue in which the microdialysis sampling will performed ultimately	[88, 89, 97, 104]
Tissue trauma	Transient local tissue trauma is caused by intradermal insertion of microdialysis probes, both in vivo and ex vivo, leading to a release of trauma-associated molecules (e.g. histamine) and changes in blood flow (in vivo). Furthermore, trauma may be induced when processing skin specimens for ex vivo studies	An equilibration period (e.g. 2 h for in vivo studies) can be included to allow wash out of trauma-induced molecules. However, the equilibration period depends on the experimental read-out and proper controls must be included if the molecule of interest is also induced by dermal trauma	[8, 69, 70, 89, 97, 105, 106]
Blood flow	The local blood flow affects wash-out/clearance of solutes and thus recovery of both exogenous or endogenous molecules at the sampling site	The axon reflex-mediated increase in blood flow is of particular importance for studies on penetration or endogenous release of small molecules as the magnitude of clearance is directly related to blood flow; consider control of flow by laser Doppler imaging Ex vivo studies may be affected by the absence of blood flow	[70, 97, 106]
Application site	Distribution of various cell types (e.g. mast cells) varies across different body sites, as does tissue thickness, which may affect the results obtained if SMD is performed in different body areas	The volar forearm is most frequently used for in vivo studies as it easily accessible, has a low frequency of hairs and presents with a flat surface area. This body site may thus serve as a “standard” when seeking to compare between different experiments	[5, 16, 56, 63–66, 72, 97, 107, 108]
Anesthetic procedure (in vivo)	Local anesthetics can be used to ease the discomfort related to the probe insertion procedure. However, the use of anesthetics (such as EMLA cream with an occlusive dressing) might affect the skin barrier and the physiological process investigated	It must be considered whether an anesthetic agent applied affects the cutaneous reactions subject to investigation. When EMLA cream is used a 40–60 min application period is recommended to minimize discomfort. Cooling of the insertion area serves as an alternative	[71, 89, 105, 109, 110]
Probe implantation depth	The implantation depth of the microdialysis probe affects which cell types will be in close vicinity of the probe, as cells are not evenly distributed through the skin layers, and may thus affect the response measured. For studies of percutaneous absorption this parameter must be controlled carefully	The probe depth should be fixed and variations must be diminished. Therefore, intradermal insertion of probes should preferably be carried out by the same skilled operator throughout a study. The precise probe depth can be assessed using ultrasound scanning	[77, 89, 97]

The members of the EAACI Task Force on SMD have developed a standard operating procedure (SOP) for performing in vivo SMD studies, which is provided in the online supplement of this report (see In vivo SMD SOP, Additional file 1).

### Ex vivo SMD

The application of SMD in studies of human ex vivo skin was first described in 1996 by Petersen et al. using the technique to measure release of histamine from skin-resident mast cells in response to intradermal injection of chemokines [74]. Since then, excised human skin has been studied by microdialysis to measure other endogenous molecules [75] and for investigations of cutaneous drug penetration [76–80]. Dermal inflammatory reactions have been studied by SMD in animal ex vivo skin [81], but this application has not yet been described for human skin specimens. Hence, human ex vivo skin has an unused potential in translational studies, as it facilitates investigations of preclinical compounds with respect to their cutaneous effects and metabolism, while reflecting the natural biological variation in contrast to studies relying on cell lines or skin substitutes. However, it is important to acknowledge that the lack of blood flow and innervation hampers studies of systemic influence on cutaneous responses. Furthermore, clearance of molecules from the tissue is also altered ex vivo, and the duration of experiments is limited by the viability of skin specimens. Similar to SMD performed in vivo, an ex vivo setup must be carefully optimized based on the target analyte(s) (see Table 1). A consensus protocol for performing ex vivo SMD studies, developed by the EAACI Task Force on SMD members, is provided in the online supplement of this report (see Ex vivo SMD SOP, Additional file 2).

## The strengths and limitations of SMD

SMD is a well validated and safe technique that has been extensively used to sample intrinsic dermal chemicals, such as mediators of inflammation, from the skin, and to deliver extrinsic substances, such as drugs, to the skin. Microdialysis has made major contributions to our understanding of dermal inflammatory disease and has driven innovative thinking in PK/PD drug studies. Still, there are limitations related to the technique that must be acknowledged and considered before using SMD to study inflammation and allergy. Table 2 summarizes some of the technique’s strengths and limitations.

**Table 2:** Strengths and limitations of SMD for the study of inflammation and allergy in the skin (see text for further information and references)

Strengths	Limitations
• Can be used with equal efficacy in both healthy and diseased skin • Allows dynamic, real-time assessment of intercellular messengers • Provides objective information on signaling pathways between resident inflammatory cells, sensory nerves and the vasculature • Used to explore the temporal and spatial variations in mediator or metabolic profiles • Probes with different MWCO allow the recovery of small molecules (e.g. histamine) away from metabolic enzymes and the recovery of larger molecules (e.g. cytokines and neuropeptides) • Use of low perfusion rates and/or the addition of colloid or lipid emulsions to the probe perfusate enhances solute recovery and limit hydrostatic fluid loss • Can be used in conjunction with other techniques, such as laser Doppler blood flux imaging and/or tissue histology in studies of dermal inflammatory and allergic reactions • Probe insertion is easy for the physician and relatively pain free, particularly when inserted under local anesthetic • Probes may be left in place for up to several days • Probes leave no scarring • Analysis platforms are continually improving e.g. development of microfluidic platforms for continuous on-line assay of dialysates	• Introduction of a microdialysis probe into the skin is a (minimally) invasive procedure necessitating appropriate controls in order to assess whether particular molecules are truly related to the disease state under investigation or have been generated as part of the tissue response to probe implantation • Despite application of local anesthetic, the insertion of microdialysis probes may be associated with mild pain • Diffusion of chemicals in the skin, particularly large molecules, is very limited. Consequently, maximum probe perfusion rates need to be low (0.1–5 µl/min) • Small recovery volumes and low concentrations of recovered chemicals make the use of assays with an appropriate sensitivity an absolute necessity • Microdialysis recovery of high-molecular-mass substances, such as cytokines and neuropeptides, has proved particularly problematic • Reduced recovery due to reduced solute bioavailability within the tissue space or to the adherence of bioactive molecules onto the material of the implanted probe • Long-term studies require the use of portable pumps, which may affect the ability of study participants to move freely depending on the duration and the anatomical site • Experienced personnel are required for optimal results (e.g. to insert probes at a consistent depth)

## Ethical considerations in SMD studies

The use of SMD in humans has been permitted through the approval of microdialysis probes by the US Food and Drug Administration (FDA) and the European Union Conformite Europeene (CE) [82].

A significant benefit of SMD is its minimally invasive nature compared to alternative tissue sampling techniques. Still, whenever research is carried out on humans or human tissue, potential ethical issues must be considered. The ethical requirements related to the use of SMD depend on the setting in which the technique is applied. In vivo studies are always subject to ethical approval from local Ethics Committees (in accordance with the Declaration of Helsinki [83]). Whether the sourcing and use of human ex vivo skin for research purposes should also be approved by an ethical committee might be a question of the anonymity status of the donor. Acquisition of fully anonymized tissue may in some countries be exempt from ethical approval, however, with the entry into force of the European General Data Protection Regulation (GDPR) the true anonymity of the donor might be brought into question.

It is advisable to contact local ethical authorities to clarify the need for ethical approval of ex vivo SMD studies and to obtain informed consent from skin donors.

## Outlook: future applications of SMD

SMD has great potential to become a standard and routinely used technique not only in experimental dermatology and allergology but also in the pharmaceutical and cosmetic industry. It provides quantifiable data of the mediators involved in the inflammatory response in situ. SMD has already been successfully applied in studies of inflammatory skin conditions including immediate hypersensitivity, urticaria, atopic dermatitis and drug hypersensitivity. Other skin diseases for which SMD can help to better characterize pathogenic mechanisms include bullous diseases, mastocytosis, autoinflammatory disorders, and allergic contact dermatitis.

As SMD can be performed in vivo as well as ex vivo, it can help to replace artificial skin models and animal studies to perform skin penetration studies in drug development. Although SMD is minimally invasive it must always be performed following ethical requirements in human research. The combination of microdialysis with advanced imaging techniques such as confocal microscopy or life imaging of the skin in 3D [84] may offer new perspectives. Clinical studies may benefit from SMD as it allows for in situ monitoring of molecules with a short in vivo half live (for example bradykinin) or mediators that are produced only locally and/or in low amounts meaning that changes may not result in noticeable alterations in plasma/serum levels. SMD offers the possibility to extract these mediators from the site where they are produced.

In addition to the recovery of mediators from the skin, SMD probes can be used to administer drugs locally and monitor cutaneous responses [6]. SMD could be applied in studies that involve special excipients to deliver active molecules into different layers of the skin such as transdermal delivery systems (laser-assisted drug delivery or micro needle patches), nanoparticles or the bicosome technology [78, 85]. Microdialysis is not restricted to the skin. Other tissues such as the heart, liver, embryonic tissue, brain or muscles have been successfully studied by microdialysis.

Current efforts to improve SMD are focused on making this technique more precise and easier to use and more sensitive. There is a need for a broad spectrum of probes and for portable syringe pumps that allow for long-term studies over several days without hospitalization. Advances in miniaturization of pumps and in microfluidics-based collection and analysis will make it even more convenient for the tested subject, particularly in extended sampling studies. Technological advances will help to improve detection thresholds and thus allow for the detection of trace amounts in even lower volumes [72, 73].

SMD is a valuable technology for research in dermatological allergology and beyond, and awareness of and further improvements in SMD will increase its use and utility in experimental and clinical studies.

## Additional files

**Additional file 1.** In vivo SMD SOP. A standard operating procedure (SOP) for sampling of soluble molecules from human skin in vivo using microdialysis—a protocol from the EAACI Task Force on Skin Microdialysis.

**Additional file 2.** Ex vivo SMD SOP. A standard operating procedure (SOP) for sampling of soluble molecules from human skin ex vivo using microdialysis—a protocol from the EAACI Task Force on Skin Microdialysis.

## Abbreviations

AD: atopic dermatitis
CGRP: calcitonin gene-related peptide
FasL: Fas ligand
GM-CSF: granulocyte-macrophage colony-stimulating factor
HPLC: high-performance liquid chromatography
IL: interleukin
IP-10: interferon-γ-induced protein 10
LTC_4_: leukotriene C4
MCP-1: monocyte chemoattractant protein 1
MWCO: molecular weight cut-off
PG: prostaglandin
PK/PD: pharmacokinetic/pharmacodynamic
RANTES: regulated on activation normal T cell expressed and secreted
SMD: skin microdialysis
SOP: standard operating procedure
SP: substance P
TNF: tumor necrosis factor
UVB: ultraviolet B
