# Supplementary material for: Skin microdialysis: methods, applications and future opportunities—an EAACI position paper
Source: Clin Transl Allergy. 2019 Apr 10;9:24. doi: 10.1186/s13601-019-0262-y (PMC6456961; doi:10.1186/s13601-019-0262-y)
Supplement: Supplementary file 1 — Additional file 1. In vivo SMD SOP. A standard operating procedure (SOP) for sampling of soluble molecules from human skin in vivo using microdialysis—a protocol from the EAACI Task Force on Skin Microdialysis. [file 13601_2019_262_MOESM1_ESM.pdf]

Standard operating procedure (SOP) for human *in vivo* skin microdialysis

A protocol from the EAACI Task Force on Skin Microdialysis

Aim

The aim of this document, which is a product of the EAACI Task Force on Skin Microdialysis, is to describe a standardized procedure for sampling of soluble molecules from human skin *in vivo* using microdialysis.
Skin microdialysis (SMD) facilitates recovery of endogenous and exogenous molecules from the extracellular compartment and has been used to study many dermal processes, including skin diseases (e.g. atopic dermatitis and psoriasis), percutaneous absorption, hypersensitivity reactions and cutaneous inflammation. The technique is approved for clinical use, however, the ethical permissions required must be in place before study participants can be enrolled.
Please refer to the corresponding position paper from the task force for a more detailed description of SMD and its applications. The position paper also features pictures of a SMD experiment (Fig. 2).

Timeline

The duration of a SMD procedure is highly dependent on the scope of the study and its setup. However, time estimates are listed for each part of the experiment below:

**A.** Preparation of collection vials[1]	*Allow for 2-3 hours depending on the number of collection vials*
**B.** Preparation of pump and microdialysis probes	*Allow for 1-2 hours*
**C.** Preparation of study participants and sampling	
- Local anesthesia (optional)	*Allow for up to 60 minutes depending on the type of anesthetic used*
- Probe insertion	*Allow for 15-30 minutes for insertion per probe*
- Microdialysis sampling	*Minutes to days depending on the setup*
**D.** Post-sampling procedure	*Allow for up to 15 minutes per probe*

Note

Make sure that you are acquainted with the equipment required before commencing SMD experiments and that you are familiar with the importance of probe orientation, as insertion of the guide cannulas will determine the outlet point, which should be easily accessible during the course of the experiment (refer to Fig.1-2 in the position paper).

---

[1] Preparation of collection vials can be performed the day before the actual SMD sampling is conducted or during priming of the microdialysis probes (depending on the number of probes and vials).

Materials *(must be available when commencing the experiment)*

Materials	Use and notes
**Anesthetic**	*Optional:* To minimize discomfort caused by probe insertion, e.g. lidocaine/prilocaine cream or cold pack[2]
**Catheter connectors**	To connect the probe inlet tubing to syringes mounted in the pump
**Collection vials**	For collection of dialysates during sampling, e.g. PCR tubes or glass capillaries[3]
**Disinfectant**	For skin disinfection, e.g. alcohol swabs
**Forceps**	For handling of probes
**Gauze**	For wrapping probes and glass capillaries/collection vials to the study participant, especially if a portable pump is used
**Gloves**	For aseptic handling
**Guide cannulas**	For intradermal insertion of microdialysis probes, e.g. 21G/23G syringe needles depending on the probe outer diameter or 25G needles with attached (crimped) probes
**Ice**	*Optional:* For stabilization of samples during sampling/prior to storage
**Marker**	To mark the treatment area and the probe insertion sites on the skin
**Microdialysis probes**	Self-made[4] or commercially available; linear or concentric. Low molecular weight cut-off (MWCO) (e.g. 2-5 kDa) for recovery of small molecules and high MWCO (e.g. 100-3000 kDa) probes for recovery of larger molecules
**Microinfusion pump**	For perfusion of microdialysis probes, with stable flow rates between 0.1-5 µl/min
**Occlusive dressing**	*Optional:* For local anesthesia. May also be used for fixation of probes
**Parafilm**	To cover collection vials in order to minimize evaporation during sampling

[2] The use of anesthetic may affect the results obtained from SMD studies by altering physiological processes such as local blood flow, neurogenic responses and skin barrier function.
[3] The collection vials must be appropriate for the study undertaken and should take into account the characteristics of the analyte. Important properties to consider when choosing the type of collection vial: Material (e.g. low adherence plastic tubes, possibly containing a stabilizing agent to diminish analyte breakdown, or glass capillaries), volume, label-ability, storage/freeze-ability, type of lid/seal, compatibility with refrigerated fraction collectors, etc.
[4] If probes are self-made, these must be sterilized in a proper manner for use *in vivo.*

**Perfusate**	For perfusion[5] (often a sterile physiological solution, but should be adapted to fit the target molecules[6])
**Ruler**	To measure the intradermal insertion length of microdialysis probes
**Saline (0.9% NaCl solution)**	For flushing of guide cannulas to remove dead cells and blood and to ease probe insertion. Can also be used for perfusion
**Scale**	To weigh collection vials
**Scissors**	To adjust the probe outlet
**Sterile paper dressing**	To cover the surface of the working area
**Surgical tape**	To fixate microdialysis probes during priming and sampling
**Syringes (flush)**	To flush probes and guide cannulas
**Syringes (pump)**	To contain the perfusate (sterile gas-tight or disposable plastic syringes compatible with the pump and volume delivery required)
**Ultrasound scanner**	*Optional:* To assess the intradermal probe depth

[5] CE labeled perfusates are commercially available.
[6] The perfusate composition should be modified based on the characteristics of the target analyte, e.g. if highly lipophilic, a lipid emulsion can be used. Additionally, when using high MWCO probes to recover large molecules, a colloid may be added to counteract fluid leakage from the probe caused by the larger pores in the microdialysis membrane.

Protocol

A) Preparation of collection vials[7] *(allow for 2-3 hours depending on the number of vials)*

Step	Procedure
1	Choose appropriate collection vials[3]
2	Label collection vials
3	Weigh individual collection vials and note the weight before sampling[8,9]
4	Adapt the outlet tubing to the collection vial (e.g. using a plastic stopper for glass capillaries or cover collection vials with Parafilm and punch a small hole for probe insertion using a needle or a lancet)

B) Preparation of pump and microdialysis probes[10] *(allow for 1-2 hours)*

Step	Procedure
1	If necessary, adjust the length of the (linear) microdialysis membrane[11]
2	Prepare the pump syringes for perfusion by filling them with perfusate[12] (e.g. Ringer-lactate + 1% human serum albumin (HSA)[13])
3	Mount the syringes in the microinfusion pump[14]
4	Gently flush the probes with perfusate (with a sufficient volume to ensure that the probe only contains perfusate, e.g. 200 µl) using a flush button on the pump or manually while following the fluid through the probe[15]
5	Confirm flow through the probe and ensure that it does not leak

---

[7] Preparation of collection vials can be performed the day before the actual SMD sampling is conducted or during priming of the microdialysis probes (depending on the number of probes and vials).
[8] The fluid recovery can be monitored by weighing collection vials before and after sampling. This is especially important when using probes with a high MWCO.
[9] Weighing and covering of collection vials may be omitted if sampling is carried out at higher flow rates and for shorter time intervals, as this decreases the risk of the sample evaporating.
[10] All work (including handling of probes) must be carried out on a sterile surface.
[11] Be careful not to damage the delicate membrane when adjusting its length (make sure to use sharp scissors).
[12] Avoid formation of bubbles in probes and syringes, especially when the perfusate contains proteins (e.g. HSA).
[13] When using high MWCO probes colloid additives should be included in the perfusate. For instance, HSA-enriched perfusates can be used for sampling of larger molecules such as cytokines, as it minimizes non-specific adsorption to probe components and acts as a carrier protein.
[14] Only calibrated microinfusion pumps should be used (the flow rates used must be validated).
[15] Be careful not to push out the central guide wire when flushing linear probes.

**6**	Connect the probe inlet tubing to the syringes mounted in the pump using catheter connectors[16]
**7**	Prime microdialysis probes (for up to 1 hour if the perfusate contains 1% HSA to minimize non-specific adsorption)[17] (see Fig. 2A in the position paper)

C) Preparation of study participants and sampling[18] (*allow for 15-30 minutes for insertion of each probe – the actual sampling may take from minutes to days*)

Step	Procedure
**1**	The study participant is rested comfortably while allowing easy access to the relevant skin area[19]
**2**	Select the study and/or control area (e.g. the volar forearm)[20]
**3**	If being used, apply local anesthetic (e.g. topical lidocaine/prilocaine cream or cold) to the relevant skin sites and remove it gently after the appropriate incubation time before disinfecting the skin area[21] (see Fig. 2B in the position paper)
**4**	Mark the probe entry and exit points (for linear probes*)* under slight tension of the anesthetized skin (e.g. placing the probes 2 cm apart[22])[23]
**5**	Insert the guide cannulas (either 21G or 23G syringe needles depending on the outer probe diameter) spanning the planned intradermal length (e.g. 2 cm)[24]. To avoid tattooing, insert the cannulas next to the marking[25] (see Fig. 2C in the position paper)
**6**	Flush each guide cannula inside the skin with sterile saline to ease insertion of the probe
**7**	Insert the primed microdialysis probes through the tip of the guide cannulas while the pump is running. Repeat for all probes (see Fig. 2D in the position paper)
**8**	Ensure that dialysate appears from the outlet tubing and gently pull out the guide cannulas, thus leaving the probe in place

[16] Be careful not to introduce bubbles in any part of the system.
[17] High MWCO probes may be tilted downwards (approximately 45°) during priming and sampling to ensure a proper flow.
[18] Gloves should be worn during the entire procedure to protect the operator, the study participant and the microdialysis membranes.
[19] Allow for free movement of the arm and encourage the study participant to visit the bathroom before the experiment is commenced. The study participant should wear loosely fitting clothing, which do not interfere with inserted probes.
[20] Make sure there are no scars, lesions or visible veins at the skin site.
[21] If lidocaine/prilocaine cream is used: Apply it under an occlusive dressing for up to 60 min.
[22] In general, probes should be placed at least 1 cm apart to avoid spillover, but the interprobe distance must be adapted to the analyte diffusibility in the tissue.
[23] For high MWCO probes make sure the exit point is located “downstream” of the entry point to avoid perfusion against gravity (which might cause fluid leakage from the probes)
[24] The insertion depth of the microdialysis probes must be kept as consistent as possible, for which reason the same operator should insert all guide cannulas throughout a study. The probe depth can be assessed using an ultrasound scanner.
[25] Pay attention to the direction when inserting guide cannulas, as this will determine the orientation and placement of the probe outlet. Furthermore, guide cannulas must be inserted evenly without penetrating the skin barrier apart from at the point of probe entry and at the exit point, if linear probes are used.

**9**	Gently fix the probe onto the skin using a piece of surgical tape placed on the inlet tubing immediately proximal to the membrane (see Fig. 2G in the position paper)
**10**	Place the collection vials at the outlet of the microdialysis probes and secure the vials using pieces of surgical tape if needed[26] (see Fig. 2I in the position paper)
**11**	Ensure that the skin sites are clean (e.g. wipe away any blood) and secure the pump to the study participant if a portable pump is used
**12**	Start SMD sampling, e.g. after performing relevant experimental procedures such as skin challenge[27,28] (see Fig. 2F in the position paper)
**13**	When the SMD sampling is completed, reweigh collection vials
**14**	Store the dialysates according to the analyte of interest (e.g. at –80 °C for subsequent protein analysis)

D) Post-sampling procedure *(allow for up to 15 minutes per probe)*

Step	Procedure
**1**	After completion of the experiment remove linear probes by cutting off at the inflow site to avoid introducing dirt and gently pull out (concentric probes can just be withdrawn from the tissue)
**2**	Wash the skin surface, clean away black dots (e.g. using ethanol) and apply a plaster if necessary
**3**	Discard materials (such as needles and probes) in accordance with local procedures

Abbreviations

HSA	Human serum albumin
MWCO	Molecular weight cut-off
SMD	Skin microdialysis

[26] If labile analytes are studied, the collection vials should be cooled during or immediately after sampling.
[27] Equilibration time may be added prior to sampling to reduce the impact of trauma from probe insertion and the effect of local anesthesia, if used.
[28] Remember to accommodate for sampling dead space in the collection protocol.
